# Effects of Bleaching Associated with Er:YAG and Nd:YAG Laser on Enamel Structure and Bacterial Biofilm Formation

**DOI:** 10.1155/2021/6400605

**Published:** 2021-12-20

**Authors:** Xiuxiu Hou, Keyong Yuan, Zhengwei Huang, Rui Ma

**Affiliations:** ^1^Department of Endodontics, Shanghai Ninth People's Hospital, Shanghai Jiao Tong University School of Medicine, Shanghai 200011, China; ^2^College of Stomatology, Shanghai Jiao Tong University, Shanghai 200011, China; ^3^National Center for Stomatology, Shanghai 200011, China; ^4^National Clinical Research Center for Oral Diseases, Shanghai 200011, China; ^5^Shanghai Key Laboratory of Stomatology, Shanghai 200011, China

## Abstract

**Objective:**

To compare the effects of bleaching associated with Er:YAG and Nd:YAG laser on enamel structure and mixed biofilm formation on teeth surfaces.

**Materials and Methods:**

Sixty-eight enamel samples were randomly divided into four groups (*n* = 17), control, Opalescence Boost only, Opalescence Boost plus Er: YAG laser, and Opalescence Boost plus Nd:YAG laser. The structure was observed using SEM after bleaching. Subsequently, the treated enamel samples were also cultured in suspensions of *Streptococcus mutans*, *Streptococcus sanguis*, *Actinomyces viscosus*, and *Fusobacterium nucleatum* (*Fn*) for 24 and 48 h. Biofilm formation was quantified by crystal violet staining, and the structure was visualized by confocal laser scanning microscopy. The data were analyzed using the Kruskal-Wallis method.

**Results:**

The enamel structure significantly changed after bleaching. There was no obvious difference in the biofilm formation after 24 h; however, after 48 hours, the amount of biofilm increased significantly. Remarkably, the amount was significantly higher on enamel bleached only, however, there was no significant difference between samples bleached with Er:YAG or Nd:YAG laser compared to the control.

**Conclusions:**

Bleaching only appeared to markedly promote biofilm formation after 48 h, and the biofilms on samples bleached with Er:YAG or Nd:YAG laser did not change significantly, showing that bleaching with Er:YAG or Nd:YAG laser can be safely applied in clinical practice.

## 1. Introduction

Tooth staining is an aesthetic problem that plagues many people. Compared to veneers and crown restorations, tooth bleaching is a conservative and noninvasive option for treating tooth staining [1]. Moreover, in-office bleaching with high concentrations of hydrogen peroxide can meet patients' demands quickly. Recently, it has been accepted that hydrogen peroxide can penetrate the tooth structure and produce free radicals that oxidize the colored organic molecules [2] [3, 4]. The reaction can also be accelerated under photochemical reactions initiated by light or laser [5].

Laser tooth bleaching began in 1996 with FDA approval of argon (488/514 nm) and carbon dioxide (10600 nm) lasers. In 2007, diode lasers (980 nm) also received FDA approval. Recently, photodynamic bleaching has been viewed as an ideal treatment option because of its shorter working time and lower postoperative hypersensitivity [6, 7]. LightWalker dual-wavelength laser from Fotona has two distinct wavelengths, including the Er:YAG (erbium: yttrium-aluminum-garnet) laser (2940 nm) and the Nd:YAG (neodymium:yttrium-aluminum-garnet) laser (1064 nm). The wavelength of Er:YAG laser is close to the water absorption peak, and the energy is mostly absorbed by the gel for heating, which reduces the damage to the hard tissue of teeth and renders the procedure safe and minimally invasive [8]. Nd:YAG laser can quickly decompose hydrogen peroxide and penetrate into the tooth to achieve a good bleaching effect.

However, clinical applications of tooth bleaching still impart many adverse effects on soft and hard tissues [9]. Studies have shown that laser-activated bleaching agents seem to be more surface-friendly than other bleaching systems [10], and laser-assisted bleaching using Er,Cr:YSGG lasers did not affect enamel [11]. In contrast, partial loss of the surface enamel can be observed after frequent KTP laser [12], and home-bleached enamel treated with Nd:YAG and Er:YAG laser exhibited some melting and recrystallized areas [13], suggesting that tooth whitening procedures using lasers damage the enamel surface more aggressively than simple peroxide treatment [14]. Such alterations to the enamel surface could also subsequently affect oral bacterial adhesion [15].

Caries is one of the most prevalent infectious diseases worldwide, and the imbalance or dysbiosis of the microbial population within the biofilm covering enamel can lead to caries [16], so it is important to study the formation of biofilms on bleached enamels [17]. At present, the effect of bleaching on the formation of bacterial biofilms is inconclusive, and studies found significant reductions in *S. mutans* populations in subgingival and supragingival plaques after bleaching [18]. However, other studies revealed that bleaching with 35% hydrogen peroxide markedly promoted *S. mutans* and *S. sanguinis* biofilm formation [15] [19]. And to our knowledge, no study has examined biofilm formation on enamel after in-office bleaching with Er:YAG or Nd:YAG laser.

As we know, the oral cavity is a complex microecological environment that contains a large number of different bacterial species, proteins, and impurities, it is difficult to replicate the real oral environment via a single kind of bacteria [17], and the biofilm formed by mixed bacteria is more similar to the oral biofilm. *Streptococcus mutans* is recognized as the most important cariogenic bacterium. Its acid production and acid resistance play important roles in the occurrence and development of caries [20]. *Streptococcus sanguis* is a pioneering colonizer, aiding in the attachment of subsequent organisms, and is a key bacterium in oral biofilm development [21]. As a smart cariogenic pathogen, *Actinomyces viscosus* can store polysaccharides and prolong acid production upon sugar deficiency [22]. *Fusobacterium nucleatum* is one of the most common Gram-negative anaerobic bacteria in dental plaques and can coaggregate with primary colonizing bacteria; thus, playing an important role in the development and maturation of dental plaques [23]. Therefore, this study intended to use the above four bacteria to simulate the formation of oral plaque biofilm.

Thus, the aim of this study was to examine enamel surface structure and biofilm formation of mixed bacteria on human enamel samples after bleaching with or without the use of Er:YAG and Nd:YAG laser. Three null hypotheses were set prior the study; H_0_1: the laser-assisted tooth bleaching treatments would not change the enamel structure compared to the conventional bleaching technique; H_0_2: there would be no differences in biofilm amount among the groups after 24 h; H_0_3: there would be no differences in biofilm amount among the groups after 48 h.

## 2. Materials and Methods

### 2.1. Specimen Preparation and Treatment

Sixty-eight enamel samples were prepared from sound permanent third molars extracted due to periodontal complications or orthodontic treatments. Samples were stored in 0.9% normal saline. The teeth were polished with pumice and a rubber cup and cut into 4mm × 4mm × 1mm sections. The upper left corner of the samples was rounded to distinguish the front and the back. Specimens without cracks observed under a stereo microscope (Leica MC170 HD, Germany) were chosen and ground flat with 400-, 600-, 1200-, and 2000-grit silicon carbide papers. Samples were then randomly divided into four groups of 17 specimens each, including the (A) control, (B) Opalescence Boost only (Opalescence Boost, USA), (C) Opalescence Boost plus Er: YAG laser treatment, and (D) Opalescence Boost plus Nd:YAG laser treatment. Samples were then treated according to the following protocols (Tables 1 and 2).

- *Control*. The group was stored in normal saline and no bleaching was performed
- *Opalescence Boost Only*. The samples received a conventional bleaching regimen, which included a one-time application of 40% hydrogen peroxide with a gel thickness of 1.5 mm, remaining on the teeth for 15 minutes following manufacturer's instructions
- *Opalescence Boost plus Er:YAG Laser Treatment*. The samples were painted with the whitening gel and irradiated with a 2940 nm Er:YAG laser (LightWalker, Fotona, Slovenia). The gel was activated with a bleaching setting of 0.4 w and 10 Hz in VLP mode (1000-microsecond pulse duration) using the R17 handpiece for 20 seconds (20 seconds per enamel specimen) at a 2 cm working distance and 5 mm spot size diameter. The gel was maintained on the specimens for 8 minutes after laser irritation. Finally, each specimen was rinsed with 5 mL sterile saline for 30 seconds
- *Opalescence Boost plus Nd:YAG Laser Treatment*. The tooth whitening gel was used in combination with a 1064 nm Nd:YAG laser (LightWalker, Fotona, Slovenia). The gel was activated with a bleaching setting of 8 w and 60 Hz in VLP mode using the R24 handpiece five times for 4 seconds each time (20 seconds per enamel specimen) at a 2 cm working distance and 6 mm spot size diameter. The gel was maintained on the specimens for 8 minutes after laser irradiation. Finally, each specimen was rinsed with 5 mL sterile saline for 30 seconds

### 2.2. Scanning Electron Microscopy

Three samples from each group were examined under SEM (Zeiss, Germany). Samples were fixed with 2.5% glutaraldehyde and then dehydrated successively with 30%, 50%, 70%, 95%, and 100% ethanol. Then, the samples were dried at a critical point. Photomicrographs were obtained using different magnifications up to 5,000× to detect any changes in surface morphology.

### 2.3. Bacterial Cultures

Frozen (-80°C) stocks of *Streptococcus mutans* (*Sm*) UA159, *Streptococcus sanguis* (*Ss*) ATCC10556, *Actinomyces viscosus* (*Av*) ATCC19246, and *Fusobacterium nucleatum* (*Fn*) ATCC25586 (provided by the Oral Microbiology Laboratory, The Ninth People's Hospital, Shanghai Jiao Tong University School of Medicine) were resuspended, transferred onto Brain heart infusion agar plates, and incubated at 37°C with 5% CO_2_ for 48 hours. A single colony was then inoculated into sterile brain heart infusion media and incubated at 37°C with 5% CO_2_ for 16 h. Then, 50 *μ*L of each bacterial solution was obtained, Gram-stained, and assessed under an optical microscope (Nikon, Japan) to verify that the bacteria were free of contamination. The optical densities of each bacterial suspension were measured at 550 nm (OD_550nm_) with a spectrophotometer, and the bacterial concentration was adjusted to about 10^5-6^ CFU/mL. The four bacterial suspensions were then mixed in equal volumes in brain heart infusion containing 1% sucrose to perform biofilm assays.

### 2.4. Biofilm Formation Assays

The sterilized specimens were added to 24-well plates and 400 *μ*L of bacterial suspensions containing 1% sucrose in BHI was dispensed onto each enamel sample. For the negative control, each group had a well that received media only, without bacteria or enamel. All samples were incubated at 37°C with 5% CO_2_ for 24 or 48 hours.

The total amount of biofilm formed (*n* = 4 for each group) was quantified using crystal violet. The bound crystal violet was extracted using 400 *μ*L of destaining solution (95% ethanol). A volume of 200 *μ*L was then transferred to a new 96-well plate. A microplate reader (Molecular Devices, USA) was used to measure the optical density of the destaining solution at 595 nm (OD_595nm_), which represented the amount of total biofilm formed. The mean optical densities of the background groups were removed from the optical density values of the crystal violet values from their respective biofilm groups.

An additional three specimens from each group were prepared with SYTO9/PI fluorescent stain (Sigma, USA) and incubated for 15 minutes in the dark. Then, the biofilm was observed using a laser confocal scanning microscope (LSM510, Zeiss, Germany). The observation conditions included an Ar laser at 488 nm, an HeNe laser at 543 nm, and a 63× oil lens. Three different sites were randomly observed on each enamel sheet. Finally, to measure the thickness of the biofilm after 48 hours.

### 2.5. Statistical Analysis

Statistical calculations were performed using SPSS version 26.0 software (SPSS Inc., Chicago, IL, USA). The amount of biofilm formed between different groups was evaluated using the Kruskal-Wallis test. Statistical significance level was at *α* = 0.05.

## 3. Results

### 3.1. SEM Observations

**Figure 1:**
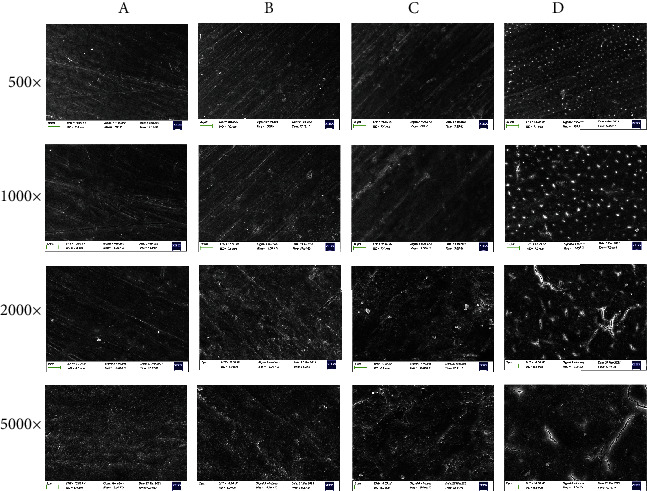
Scanning electron microscopy (SEM) images showing the structure of enamel surfaces (magnification up to 5,000×). (a) Control; (b) Opalescence Boost only; (c) Opalescence Boost with Er:YAG laser; (d) Opalescence Boost with Nd:YAG laser.

The SEM observations were recorded to compare the structure between the groups at a magnification of up to 5,000× (Figure 1). Scratches caused by strong polishing were observed at 1,000× magnification. At 5,000× magnification, the surface of the control samples revealed no signs of enamel damage, while bleached enamels showed varying degrees of surface alterations, including porosities and cracks. The surfaces that underwent bleaching only showed partial damage with notable enamel dissolution and the presence of tomes processes. Unlike the bleached only, samples that underwent Er:YAG laser-activated bleaching exhibited noticeable losses in the integrity of the enamel surface. Additionally, enamel erosion was also indicated by the shearing of enamel rods. In those samples, the interprismatic spaces were predominantly damaged, and the loss of the interprismatic substance was evident. In the samples treated with Nd:YAG, the loss of integrity of the enamel was more aggressive than control, and surface fractures were observed.

### 3.2. Biofilm Formation

**Figure 2:**
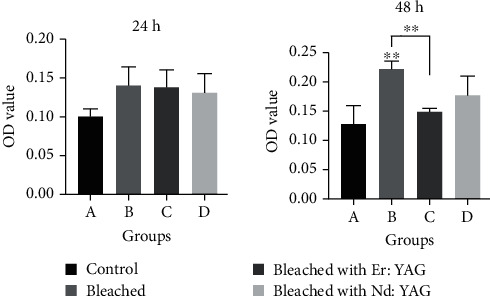
Crystal violet assays with optical density values at 595 nm (OD595 nm) representing the amount of mixed biofilms on the enamel. The error bars represent the standard deviation of measurements for 4 samples in each group. Asterisk (∗) represents significant differences (*p* < 0.05) according to Kruskal-Wallis tests. (a) Control; (b) Opalescence Boost only; (c) Opalescence Boost with Er:YAG laser; (d) Opalescence Boost with Nd:YAG laser.

From biofilm formation assays, the OD values of mixed biofilms from all four groups after 24 hours were low, and no differences were observed between groups. However, after 48 hours, the OD values increased and the levels of biofilm formation on the enamel samples were significantly different. Bleaching only using 40% H_2_O_2_ led to the highest level of biofilm formation, while there was no significant difference in biofilm formation between the other groups (Figure 2).

**Figure 3:**
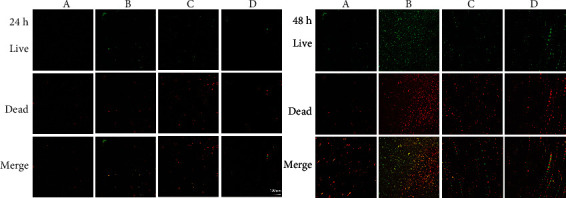
Confocal laser scanning microscope (CLSM) images showing the structure of biofilms on enamel surfaces (63×). (a) Control; (b) Opalescence Boost only; (c) Opalescence Boost with Er:YAG laser; (d) Opalescence Boost with Nd:YAG laser.

The CLSM images (Figure 3) revealed the structures of biofilms in each treatment group, with the bacteria being stained green (live bacteria) or red (dead bacteria). For the 24 hours' biofilm, similar structures and thin biofilms were observed on the enamel surfaces in all groups. For 48 hours' biofilms, the number of cells in the unbleached group was lower than that of the bleached groups, while the bleached only group contained the most bacteria. Similar biofilm structures were observed on the enamel surfaces of samples bleached and treated with Er:YAG or Nd:YAG laser. And the bleached only group had the thickest biofilm after 48 hours (Table 3), which is consistent with the results observed by confocal microscopy.

## 4. Discussion

With the increased popularity of dental bleaching and the introduction of newer bleaching approaches, concerns have been raised about the efficacy, side effects, and risk of caries associated with such approaches [24]. Therefore, the aim of this study was to evaluate the enamel surface structures and biofilm formation after tooth bleaching using two new types of laser-activated approaches.

On the basis of the results reported in the current study, Ho1 stating that the laser-assisted tooth bleaching treatments would not change the enamel structure compared to the conventional bleaching technique was rejected. Our results demonstrated that the surface of the control samples revealed no evidence of enamel damage, while bleached enamel samples exhibited varying degrees of surface changes, including increased porosities and cracks.

Studies have shown that the structure and morphology of enamel can be affected by bleaching with laser irradiation [25]; however, the findings of such studies have not been consistent [4, 12, 14, 26, 27]. In our study, partial exposure of enamel rods after bleaching with Er:YAG laser was similar to the finding bleached with Er:YAG laser with 6% or 35%H_2_O_2_ [8]. Er:YAG laser cannot penetrate into enamel deeply, but can cause microblasting of inorganic substances and ablation of organic substances, so the enamel becomes molten and the enamel rods were exposed. Surface fracture was observed in the group receiving Nd:YAG laser treatment because Nd:YAG laser can quickly penetrate into the tooth and destroy enamel. But other studies suggested that the surface treated by Er:YAG laser showed irregular and microporous surface with flake pattern [13], and the surfaces treated with Nd:YAG lasers exhibited some melting and recrystallized areas [11]. Such differences could be due to the variations in different conditions, such as the tooth substrates (human or bovine), concentration and pH of bleaching gel, laser wavelength, and irradiation time, among other variables. However, it is unclear how enamel surface changes affect biofilm formation or other clinical parameters after bleaching using lasers.

Dental plaque biofilm refers to a clump of bacteria that accumulates on the tooth surface or other hard tissues and cannot be washed away by moderate water, and the dysbiosis of dental biofilms adhering to tooth surfaces can lead to caries [28]. It is well known that plaque biofilms rapidly formed within 24 hours and mature within 48 hours after the tooth surface was cleaned. Therefore, this study simulated the clinical procedures recommended by the manufactures and tested for the formation of mixed biofilms after 24 hours and 48 hours of culture.

At 24 hours, the OD values were relatively low, there was no difference between each group, and CLSM revealed a high proportion of dead bacteria due to the bactericidal effect of hydrogen peroxide, so the H_0_2 was accepted. While H_0_3 was rejected and after 48 hours of culture, an increase in biofilm formation was found on bleached enamel samples compared to the control samples. Additionally, samples treated by bleaching only with 40% H_2_O_2_ led to the highest levels of biofilm formation, which was consistent with the report that bleaching leads to increased microbial adhesion on the enamel surface [29]. The increased attachment on bleached enamel may be due to the porosities and cracks on the enamel surface. However, biofilm formation on enamel bleached using lasers was lower than that on enamel bleached only, suggesting that laser bleaching has bactericidal or antibacterial effects. Although previous studies also suggested that laser irradiation may have a bactericidal effect [30, 31], it is not clear whether such photochemical toxicity exists when biofilm formation is carried out following laser irradiation. Thus, it is necessary to further evaluate the changes to enamel surface roughness and mineral content following laser treatment.

As the purpose of this study was to compare the effects of Er:YAG and Nd:YAG laser-activated bleaching systems, both laser sources were used with bleaching agents containing 40% H_2_O_2_ to eliminate any discrepancies caused by the bleaching gels. In addition, because the strains and culture conditions used in this study were different from those in oral environments, the process, time, and structure of biofilms were different than those found under real circumstances. Therefore, additional experimental techniques and methods are needed to further reveal the effects of bleaching using different lasers on the formation of plaques on enamel surfaces.

## 5. Conclusions

The enamel surface structure significantly changed after bleaching with or without laser treatment. Bleaching only appeared to markedly promote biofilm formation after 48 hours, while biofilms formed on samples that underwent bleaching with Er:YAG or Nd:YAG laser did not change significantly, suggesting that bleaching with Er:YAG or Nd:YAG laser failed to promote the occurrence of caries and can be safely applied in clinical practice.

## Figures and Tables

**Table 1:** Bleaching program in different treatment groups.

Group	Bleaching gel	Bleaching time	Er:YAG laser	Nd:YAG laser
Control	—	0	—	—
Opalescence Boost only	Opalescence Boost 40%PF	15 min	—	—
Opalescence Boost plus Er: YAG laser	Opalescence Boost 40%PF	8 min	20 s	—
Opalescence Boost plus Nd:YAG laser	Opalescence Boost 40%PF	8 min	—	5∗4 s

**Table 2:** Fotona dual-wavelength laser used for teeth whitening data.

Program	Laser source	Pulse width	Frequency	Spot diameter	Power	Hand tools
Bleaching	Er:YAG laser	VLP	10 Hz	5 mm	0.4 W	R17
Bleaching	Nd:YAG laser	VLP	60 Hz	6 mm	8 W	R24

**Table 3:** Biofilm thickness in each group after 48 hours, *n* = 3.

Group	A	B	C	D
Biofilm thickness (*μ*m)	7.67 ± 3.21	43.3 ± 2.89	12 ± 1	14.67 ± 3.05

## Data Availability

The figures data used to support the findings of this study are included within the article.
